# SIMOA-based analysis of plasma NFL levels in MCI and AD patients: a systematic review and meta-analysis

**DOI:** 10.1186/s12883-023-03377-2

**Published:** 2023-09-18

**Authors:** Hadi Sahrai, Ali Norouzi, Sina Hamzehzadeh, Alireza Majdi, Rana Kahfi-Ghaneh, Saeed Sadigh-Eteghad

**Affiliations:** 1 Student Research Committee, Tabriz University of Medical Sciences, Tabriz, Iran; 2 Exp ORL, Department of Neuroscience, Leuven Brain Institute, KU Leuven, Louvain, Belgium; 3 Neurosciences Research Center, Tabriz University of Medical Sciences, Tabriz, Iran

**Keywords:** Alzheimer’s disease, Mild cognitive impairment, Serum, Neurofilament light chain, Single molecule array assays

## Abstract

**Background:**

The single-molecule array assay (SIMOA)-based detection of neurofilament light (NFL) chain could be useful in diagnosing mild cognitive impairment (MCI) and Alzheimer’s disease (AD). This meta-analysis aimed to evaluate the circulating concentration of NFL in AD and MCI patients compared with healthy controls using the SIMOA technique.

**Methods:**

To this end, Google Scholar, PubMed, Scopus, Web of Science, and the reference lists of relevant articles were systematically searched for studies reporting serum NFL chain levels in healthy controls, MCI, and AD patients. Appropriate statistical methods were employed to achieve the study purpose.

**Results:**

Fifteen eligible studies including 3086 patients were pooled out of a total of 347 publications. Fixed effect model analysis showed that NFL chain level was significantly higher in the serum of patients with MCI (0.361 SMD, 95% CI, 0.286–0.435, *p* = 0.000, *I*^2^ = 49.179) and AD (0.808 SMD, 95% CI, 0.727–0.888, *p* = 0.000, *I*^2^ = 39.433) compared with healthy individuals. The analysis also showed that the NFL chain levels in plasma were significantly different between patients with MCI and AD (0.436 SMD, 95% CI, 0.359–0.513, *p* = 0.000, *I*^2^ = 37.44). The overall heterogeneity of the studies was modest.

**Conclusions:**

This study highlights the potential of serum NFL chain detected using SIMOA in differentiating MCI, AD, and healthy controls.

## Background

Alzheimer’s disease (AD) is widely recognized as the most common etiology of dementia [1] and is currently ranked as the sixth most prevalent cause of mortality in the United States. The global prevalence of AD is expected to rise to 135 million individuals by the year 2050 [1].

There is currently no definitive diagnostic test or biomarker for the disease, which means that diagnosis often involves ruling out other causes of cognitive decline [2]. Several biomarkers have been identified that can potentially be used for diagnosing AD in its early stages. The four main biomarkers found in cerebrospinal fluid (CSF), i.e., amyloid beta (Aβ)_1–42_, Aβ_42/40_ ratio, Tau, and phosphorylated-Tau (p-Tau)_181_, are reliable for supporting AD diagnosis as they indicate the hallmark AD pathologies of amyloidosis and neurodegeneration [3]. Although these CSF biomarkers are reliable for supporting AD diagnostics, the process of collecting CSF can be inconvenient for subjects and may cause procedural efforts. This prevents their use as a screening item in initial, asymptomatic subjects and makes repetitive monitoring of the disease progression challenging. Therefore, there is a significant necessity to develop blood-based markers that can provide targeted and fairly noninvasive screening tests in the right context of clinical application [4].

Evidence suggests that the neurofilament light chain (NFL) levels, a marker of cytoskeletal protein that rises in CSF and serum following neuroaxonal impairment, increase in individuals with AD [5]. In that line, according to a recent report by Mattsson et al., it was found that plasma NFL can be reliably regarded as a neurodegeneration biomarker in AD [6]. Nevertheless, NFL is not specific to AD, and its concentration similarly increases in other forms of neurodegenerative diseases including vascular and frontotemporal dementias [7]. The insensitivity of classical ELISA methods in accurately detecting trace NFL levels in circulation is another issue that needs to be addressed in the field [8].

Recently, ultrasensitive single molecule array assays (SIMOA) have been used to measure CSF and blood biomarkers. SIMOA technique can measure low concentrations of Aβ, p-Tau, and NFL in blood samples, reflecting those levels measured in CSF [9]. Therefore, accurate measurement of NFL levels in plasma using ultra-sensitive technics such as SIMOA could be a significant step in the early diagnosis of AD [10].

Here, this meta-analysis aimed to evaluate the diagnostic accuracy of plasma NFL levels measured by SIMOA in distinguishing between AD, MCI, and healthy controls.

## Methods

### Search strategy

The following databases were searched for published articles in English from inception until February 2023: Scopus, Google Scholar, Web of Science, and PUBMED. Our search used the following keywords: Alzheimer’s disease, AD, mild cognitive impairment, MCI, single molecule array, SIMOA, plasma neurofilament light, and NFL as follows: ((((("Alzheimer"[Title/Abstract]) OR ("Alzheimer’s"[Title/Abstract])) OR ("AD"[Title/Abstract])) AND (("neurofilament light") OR ("Nfl"))) AND (("MCI"[Title/Abstract]) OR ("Mild cognitive impairment"[Title/Abstract]))) AND ((("Serum") OR ("Plasma")) OR ("Blood")). In addition, we searched references of selected articles to identify potentially-related studies. The results were reported based on Preferred Reporting Items for Systematic Reviews and Meta-Analyses (PRISMA) statement criteria [11]. Two investigators independently searched the databases and in case of any inconsistencies, a senior researcher judged the articles against the inclusion and exclusion criteria, and approved the final list of articles.

### Study selection

Studies were considered for inclusion if they met all of the following criteria: 1) Articles reported plasma NFL levels in controls, AD, and MCI patients; 2) Plasma NFL levels were measured by SIMOA; 3) Human studies without restriction in age range and sample size; 4) Clinical studies, longitudinal studies such as cohort or case–control, retrospective or prospective; 5) Studies written in English; 6) Availability of information for each study, including sample size and diagnostic criteria for AD and MCI.

After article identification and review, studies without sufficient data and/or those focusing on other types of diseases (e.g., Parkinson’s disease, Down syndrome, frontotemporal dementia, and vascular dementia) were excluded. Additionally, this study excluded duplicate articles, reviews, case reports, and irrelevant papers.

### Data extraction and critical appraisal

Two researchers independently extracted the data from each study, and any disagreement was reviewed by a third author if it was needed. From each included study, the following data were extracted: first author, publication year, age of patients, number of participants, plasma NFL levels, and measuring method of the biomarker. Quality assessment of the studies was accomplished by Joanna Bridge Institute (JBI) critical appraisal tools for cross-sectional, cohort, and case–control studies.

### Statistical analysis

The Comprehensive Meta-Analysis Software (CMA) software version 2.0 was used to analyze the data. All data were stated as mean ± standard deviation (SD). Meta-analysis was used if three or more articles assessed a comparable intervention by the fixed-effects model, as their heterogeneity was rather low [12]. The standardized mean difference (SMD) was used to compare the means of the groups in each publication. The *I*^2^ statistic was found not to be suitable for evaluating the variability of the effect size in our data, so we did not use it to report heterogeneity. We believe that its intended purpose does not include measuring the extent of effect size differences, and it cannot provide this information unless the *I*^2^ value is zero. Instead, we utilized the prediction interval to assess variability (heterogeneity) in our data [13, 14]. Publication bias was calculated using funnel plot and trim and fill analysis which allows the estimation of an adjusted meta-analysis estimate in the presence of publication bias [15]. A *p*-value of less than 0.05 was considered statistically significant.

## Results

**Fig. 1:**
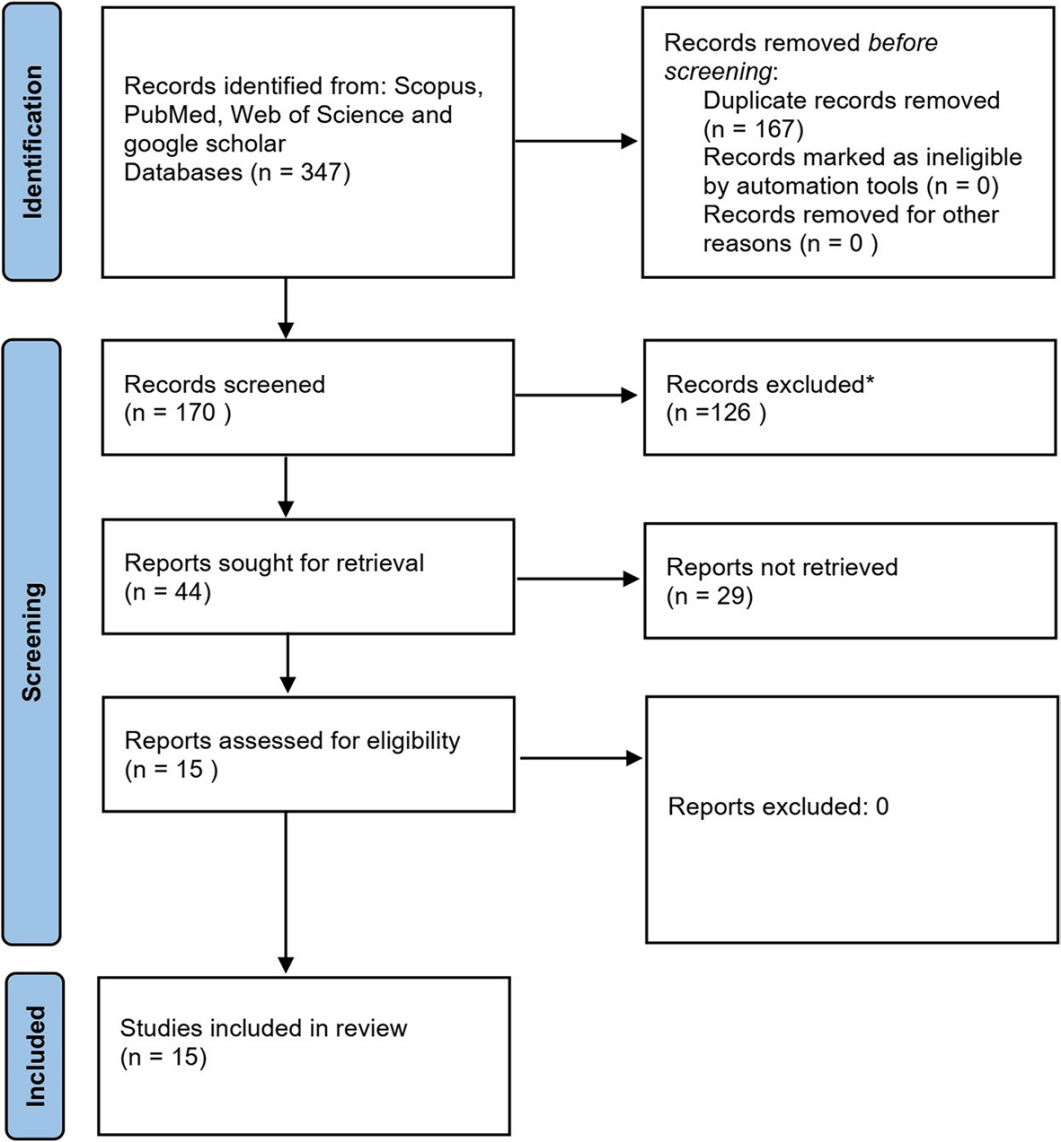
PRISMA flow diagram. Literature selection summary based on the PRISMA guidelines depicting the number of inclusions and exclusions from the initial search. * Review articles, insufficient NFL data, irrelevant articles, and other types of dementia

This study evaluated the results emerging from 15 publications. All publications assessed the relationship between NFL plasma concentrations and AD and/or MCI diagnosis. The study selection process is depicted in Fig. 1. In the first step, we found 347 articles from different databases. After removing the duplicates, 170 studies were screened based on title and abstract. Then, of the remaining 44 studies, 29 studies were excluded from full-text evaluations. Accordingly, we assessed the remaining studies based on the eligibility criteria of this systematic review. Fifteen out of 44 studies met the eligibility criteria and were included in our systematic review and meta-analysis.

### Demographic features

A total number of 4625 patients were included in the systematic review and quantitative meta-analysis. 1439 participants (31.11%) were diagnosed with MCI, while 1420 (30.70%) had AD. 1766 (38.18%) participants were evaluated as controls of the meta-analysis. Additional information of each study is presented in Table 1.

**Table 1:** Characteristics of the included studies in the systematic review and meta-analysis

**Study**	**Year**	**Participants**	**Type of study**	**Gender (M/F)**	**MMSE**	**Age**	**Education (Years)**	**APOE ε4 status, pos./neg. (% pos.)**	**Outcome**
Simren et al. [16]	2020	318	Cross-sectional	C: 46/53 MCI: 51/65 AD: 40/63	C: 29.07 ± 1.26 MCI: 27.21 ± 1.82 AD: 21.07 ± 4.42	C: 73 ± 6.14 MCI: 74.47 ± 5.89 AD: 76.35 ± 5.76	C: 11.23 ± 4.8 MCI: 8.97 ± 4.28 AD: 7.82 ± 3.66	C: 31/99 (31.3%) MCI: 39/107 (36.4%) AD: 58/103 (56.3%)	Plasma NFL level is increased in AD and MCI groups compared to healthy controls. NFL could be a diagnostic factor due to AD and MCI group changes
Gerards et al. [17]	2022	111	Cross-sectional	MCI: 33/24 AD: 24/30	MCI: 27 ± 2.1 AD: 23 ± 3.6	MCI: 69.1 ± 9.7 AD: 74.3 ± 8.2	C: NM MCI: 13 ± 3 AD: 12 ± 2	NM	Measuring plasma NFL using SIMOA could be a diagnostic factor in AD and MCI groups
Gleerup et al. [18]	2021	115	Cross-sectional	C: 11/6 MCI: 25/22 AD: 22/29	C: 28.9 ± 0.8 MCI: 26.8 ± 3.2 AD: 22.9 ± 4.3	C: 68.4 ± 8.3 MCI: 71.1 ± 8.2 AD: 72.7 ± 7.5	NM	NM	There is no correlation between the level of saliva NFL and plasma NFL measured by SIMOA in AD, MCI, and healthy control groups
Lewczuk et al. [19]	2018	110	Cross-sectional	C: 22/19 MCI: 10/25 AD: 13/21	C: 29.3 ± 0.9 MCI: 26.7 ± 2.1 AD: 21.2 ± 3.4	C: 52.5 ± 13.1 MCI: 71.3 ± 8.4 AD: 70.8 ± 7.6	NM	NM	Plasma NFL is increased in AD compared to the control group, and plasma NFL could be used as a screening biomarker between groups with cognitively declined function
Lin et al. [20]	2018	234	Cross-sectional	C: 31/28 MCI: 27/29 AD: 56/63	C: 27.8 ± 2.1 MCI: 26.4 ± 2.3 AD: 18.6 ± 6.2	C: 77.0 ± 6.2 MCI: 77.3 ± 5.1 AD: 76.0 ± 5.6	C: 12.4 ± 5.0 MCI: 9.5 ± 4.8 AD: 11.0 ± 3.7	C: 7/59 (12.1%) MCI: 13/56 (23.2%) AD: 42/119 (35.3%)	Plasma NFL may be a potential biomarker in diagnosing AD; NFL is also associated with cognitive status and cognitive function
Sugarman et al. [21]	2020	579	Cross-sectional	C: 89/149 MCI: 77/108 AD: 88/68	C: 29.39 ± 0.91 MCI: 28.20 ± 1.67 AD: 21.11 ± 6.17	C: 72.38 ± 7.69 MCI: 74.99 ± 7.24 AD: 76.74 ± 8.12	C: 16.56 ± 2.54 MCI: 15.51 ± 2.74 AD: 14.95 ± 2.95	C: 77/235 (32.8%) MCI: 59/181 (32.6%) AD: 88/153 (57.5%)	Plasma NFL is a potential biomarker in diagnosing AD and is also very sensitive to cognitive status and decline
Zhou et al. [22]	2017	578	Cross-sectional	C: 106/87 MCI: 133/65 AD: 97/90	C: 29.1 ± 0.99 MCI: 26.9 ± 1.8 AD: 23.3 ± 2.1	C: 75.7 ± 4.9 MCI: 74.5 ± 7.4 AD: 75.5 ± 7.4	C: 16.0 ± 2.8 MCI: 15.8 ± 3.0 AD: 14.7 ± 3.1	NM	Plasma NFL is not an accurate biomarker for diagnosing the early stages of AD
Hall et al. [23]	2021	546	Cross-sectional	C: 87/328 MCI: 35/63 AD: 15/18	C: 26.97 ± 2.59 MCI: 23.92 ± 3.65 AD: 16.98 ± 5.87	59.22 ± 6.97 65.61 ± 8.47 73.727 ± 8.80	C: 8.353 ± 4.30 MCI: 6.370 ± 3.99 AD: 4.818 ± 4.67	NM	Plasma NFL could be an early diagnostic factor in differentiating MCI and healthy controls in the Mexican–American population
Wu et al. [24]	2021	428	Cross-sectional (participants recruited from 2 cohorts)	C: 45/76 MCI: 68/80 AD: 68/91	C: 29.16 ± 1.12 MCI: 26.33 ± 2.24 AD: 16.66 ± 6.73	C: 69.3 ± 7.1 MCI: 69.7 ± 8.8 AD: 66.9 ± 9.9	C: 11.1 ± 3.8 MCI: 11.0 ± 3.9 AD: 8.5 ± 4.4	C: 13/121 (10.7%) MCI: 58/148 (39.2%) AD: 82/159 (51.6%)	The level of plasma NFL is a reliable diagnostic factor in AD patients in the Chinese population
Shim et al. [25]	2022	99	Cross-sectional	MCI: 6/31 AD: 17/45	MCI: 21.86 ± 3.61 AD: 14.94 ± 5.51	MCI: 77.14 ± 6.09 AD: 79.39 ± 6.66	C: NM MCI: 7.46 ± 4.93 AD: 5.92 ± 4.76	C: NM MCI: 11/34 (32.4%) AD: 13/46 (28.3%)	Plasma NFL level could be used as a possible biomarker in diagnosing AD, and the NFL level is also associated with cognitive decline
Mattsson et al. [6]	2017	570	Prospective Case–control	C: 106/87 MCI: 132/65 AD: 94/86	C: 29.1 ± 1 MCI: 26.9 ± 1.8 AD: 23.2 ± 2.1	C: 75.9 ± 4.9 MCI: 74.7 ± 7.5 AD: 75.3 ± 7.3	C: 16.0 ± 2.9 MCI: 15.8 ± 3.0 AD: 14.7 ± 3.1	C: 50/193 (25.9%) MCI: 103/197 (52.3%) AD: 123/180 (68.3%)	Plasma NFL level is elevated in AD patients as compared to the control group. Hence, plasma NFL is correlated with cognitive decline, and plasma NFL is an accurate biomarker in diagnosing AD
Parvizi et al. [26]	2022	167	Cross-sectional	C: 20/24 MCI: 34/29 AD: 24/36	C: NM MCI: 26.66 ± 2.27 AD: 19 ± 6.83	C: 62.16 ± 10.49 MCI:69 ± 14.03 AD: 69.36 ± 12,53	NM	NM	Plasma NFL is a predictive factor in diagnosing AD
Wang et al. [27]	2022	Cohort 1: 40	Cohort	C: 11/9 AD: 8/12	C: 28.83 ± 1.59 AD: 15.25 ± 6.98	C: 56.58 ± 4.98 AD: 56.58 ± 4.18	C: 10 ± 2.39 AD: 9.08 ± 0.59	C: 1/20 (5%) AD: 6/30 (30%)	Plasma NFL changes was significant in AD compared to healthy controls
Wang et al. [27]	2022	Cohort 2: 40	Cohort	C: 9/11 MCI: 7/13	C: 28.5 ± 2.39 MCI: 25.41 ± 2.59	C: 56.91 ± 8.17 MCI: 62.41 ± 11.36	C: 9.25 ± 5.38 MCI: 9.66 ± 3.19	C: 2/20 (12%) MCI: 7/20 (44%)	MCI group had a higher plasma NFL concentration compared to healthy controls
Frank et al. [28]	2022	569	Cross-sectional	C: 87/148 MCI: 86/67 AD: 76/105	C: 29.39 ± 0.91 MCI: 28.20 ± 1.68 AD: 21.12 ± 6.21	C: 72.38 ± 7.69 MCI: 74.96 ± 7.25 AD: 76.82 ± 8.13	C: 16.56 ± 2.54 C: 15.52 ± 2.77 AD: 14.95 ± 2.95	C: 77/235 (33%) MCI: 60/181 (33%) AD: 89/153 (58%)	In AD diagnosis, elevated plasma NFL concentration was correlated with a higher conditional odds ratio. In contrast, NFL concentration was not associated with an MCI diagnosis
Asken et al. [29]	2022	Cohort1: 50	Cross-sectional	NM	NM	NM	NM	NM	Plasma NFL concentration in MCI was lower compared to control group
Asken et al. [29]	2022	Cohort2: 71	Cross-sectional	NM	NM	NM	NM	NM	AD patients had lower level of NFL compared to MCI patients

### Characteristics of the included studies

Fifteen studies met the inclusion criteria. The number of participants ranged from 40 to 579. The mean age of participants in the AD group ranged from 56.58 ± 4.18 to 79.39 ± 6.66, in the MCI group ranged from 62.41 ± 11.36 to 77.3 ± 5.1, and in control group ranged from 52.5 ± 13.1 to 77.0 ± 6.2.

The reported diagnosis criteria for AD were as follows: Diagnostic and Statistical Manual of Mental Disorders, Fourth Edition, (DSM-IV) [30], National Institute of Neurological and Communicative Disorders and Stroke–Alzheimer’s Disease and Related Disorders Association (NINCDS-ADRDA), or National Institute on Aging-Alzheimer’s Association (NIA-AA) criteria [31]. Also clinical/cognitive assessments for AD patients were done using following tests: Mini-Mental State Examination (MMSE), Clinical Dementia Rating (CDR), Alzheimer Disease Assessment Scale–cognitive subscale (ADAS-Cog 11), Wechsler Memory Scale (WMS) logical memory II, Trail-Making test part B (TMT-B), Wechsler Adult Intelligence Scale–Revised (WAIS-R) digit symbol substitution test.

Also, the diagnosis criteria/tests used for MCI patients were Petersen criteria for MCI [32], NINCDS-ADRDA criteria, NIA-AA criteria, ADAS-Cog 11, MMSE, CDR, WMS logical memory II, TMT-B and WAIS-R digit symbol substitution test.

Of fifteen studies, thirteen studies were cross-sectional, one study was cohort and one study was case–control. Additional information is reported in Table 1.

### NFL plasma concentration in MCI vs control

**Fig. 2:**
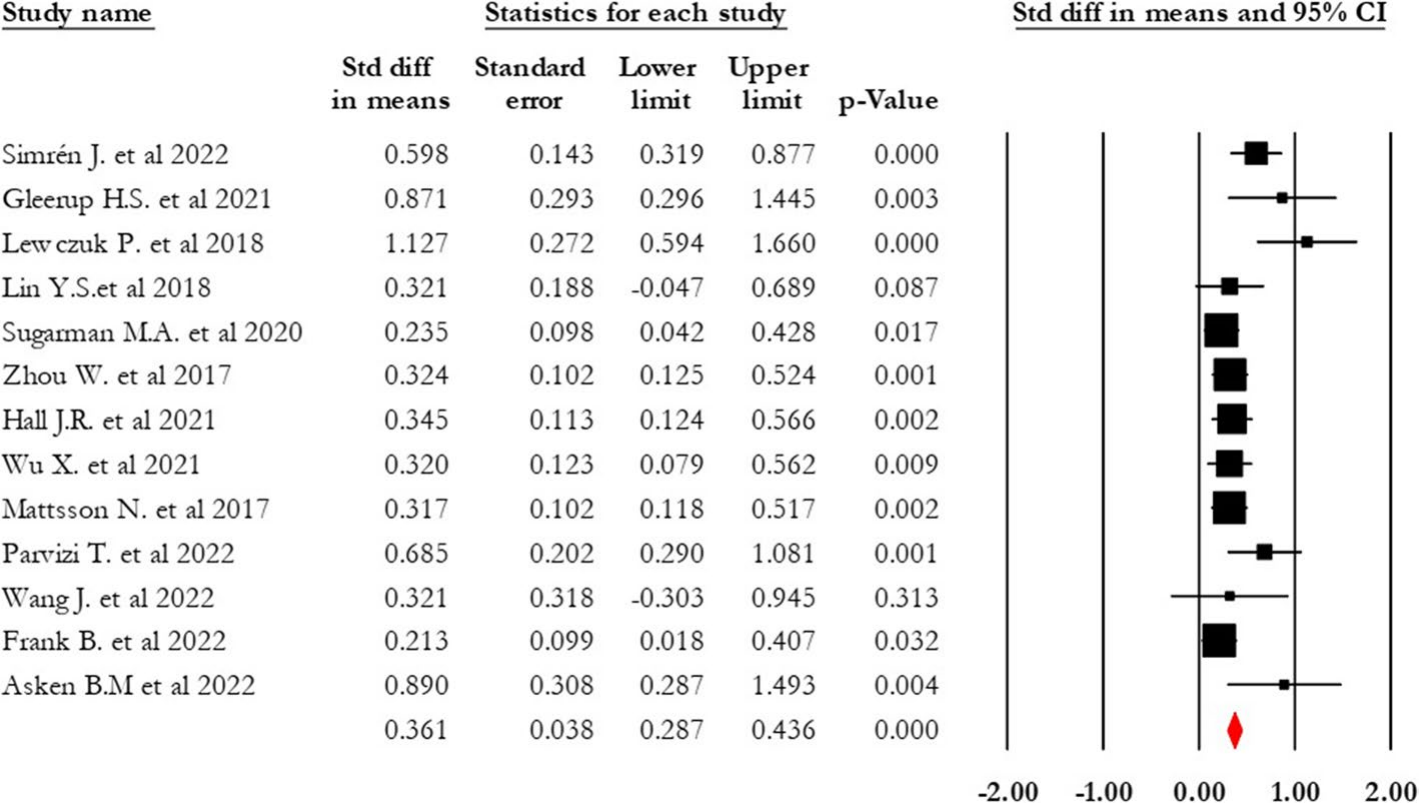
Forest plot of standardized mean difference (SMD) in MCI-control. The red square shows the overall pooled effect. Black squares indicate the SMD in each study. Horizontal lines represent a 95% confidence interval (CI)

Analysis of 13 articles revealed that the serum NFL level was significantly higher in the MCI group compared with the control group (0.361 SDM, 95% CI 0.286–0.435, *p* = 0.000, I2 = 49.179) (see Fig. 2). Trim and fill analysis showed the absence of two studies in the MCI-control comparison. Upon adjusting SMD, the values changed to 0.337 (95% CI 0.264–0.410).

### NFL plasma concentration in AD vs control

**Fig. 3:**
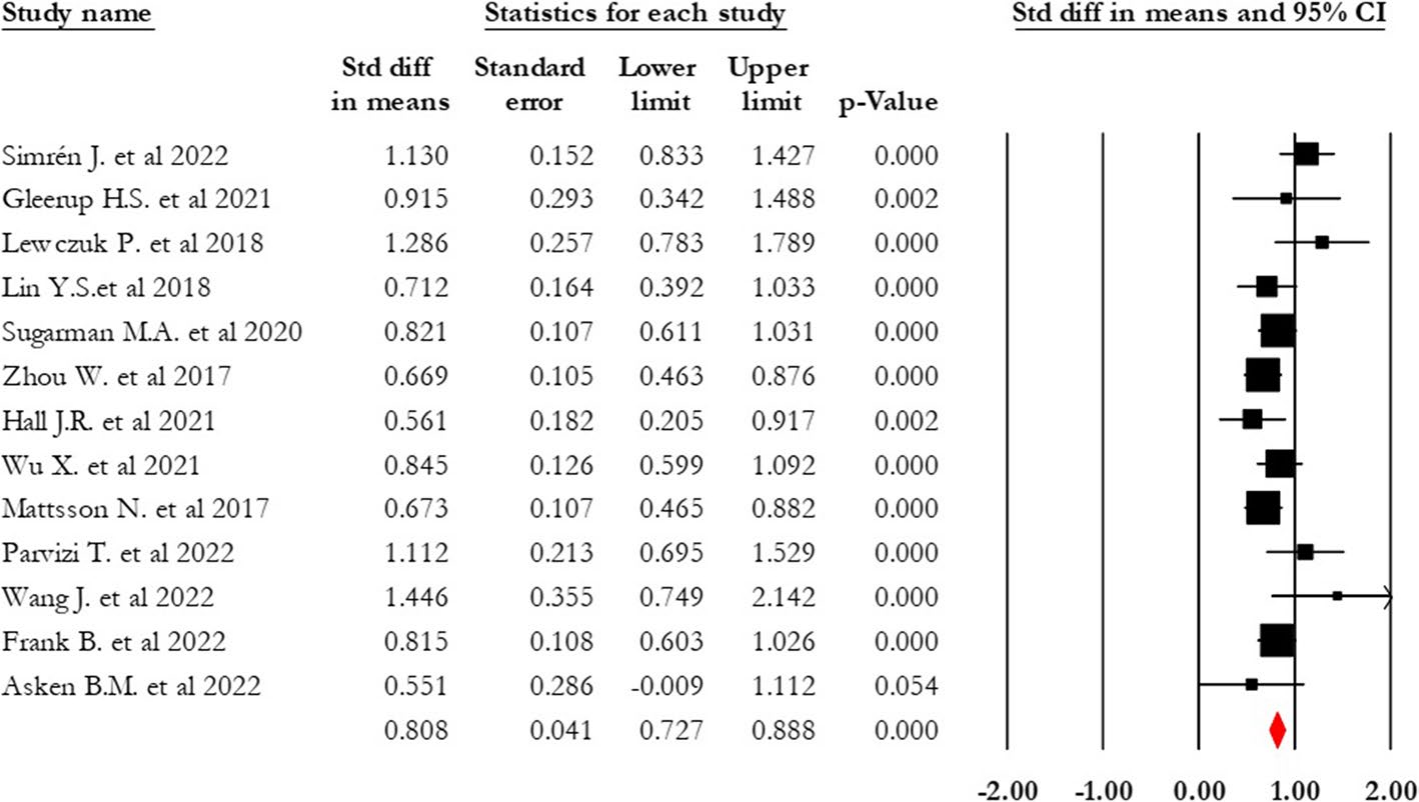
Forest plot of standardized mean difference (SMD) in AD-control. The red square shows the overall pooled effect. Black squares indicate the SMD in each study. Horizontal lines represent a 95% confidence interval (CI)

The AD group exhibited a significantly higher serum NFL level than the control group (0.808 SDM, 95% CI 0.727–0.888, *p* = 0.000, *I*^2^ = 39.433) (see Fig. 3). Trim and fill analysis identified two missing studies in the AD-control comparison. Upon adding these publications, the adjusted estimate of average efficacy in AD control decreased to 0.786 (95% CI 0.707–0.865).

### NFL plasma concentration in MCI vs AD

**Fig. 4:**
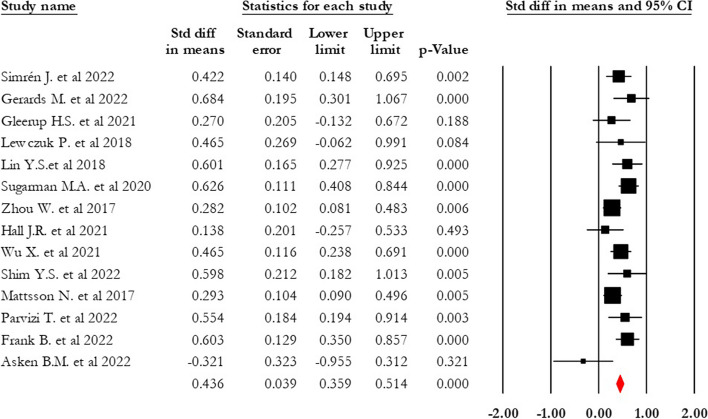
Forest plot of standardized mean difference (SMD) in AD-MCI. The red square shows the overall pooled effect. Black squares indicate the SMD in each study. Horizontal lines represent a 95% confidence interval (CI)

Through analysis of 14 articles, we observed that the serum NFL level was significantly higher in the AD group than in the MCI (0.436 SDM, 95% CI 0.359–0.513, *p* = 0.00, *I*^2^ = 37.44) (see Fig. 4). Trim and fill analysis indicated the presence of one missing study in the MCI-control comparison. After adjusting SMD, the value changed to 0.425 (95% CI 0.350–0.501).

### Risk of bias across studies

The JBI critical appraisal tool for cross-sectional, cohort, and case–control studies was utilized to assess the risk of bias in the included studies. The tool indicated no significant risk of bias across the cross-sectional, case–control, and cohort studies included in this analysis (see Tables 2, 3 and 4, respectively).

**Table 2:** The JBI critical appraisal tool for cross-sectional studies

Study name	Q1	Q2	Q3	Q4	Q5	Q6	Q7	Q8	Yes %	Risk of bias
Hall et al.	Yes	Yes	Yes	Yes	Yes	Yes	Yes	Yes	100%	Low
Simren et al.	Yes	Yes	Yes	Yes	Yes	Yes	Yes	Yes	100%	Low
Gerards et al.	Yes	Yes	Yes	Yes	Yes	Yes	Yes	Yes	100%	Low
Shim et al.	Yes	Yes	Yes	Yes	Yes	Yes	Yes	Yes	100%	Low
Gleerup et al.	Yes	Yes	Yes	Yes	Yes	Yes	Yes	Yes	100%	Low
Lewczuk et al.	Yes	Yes	Yes	Yes	Yes	Yes	Yes	Yes	100%	Low
Lin et al.	Yes	Yes	Yes	Yes	Yes	Yes	Yes	Yes	100%	Low
Sugarman et al.	Yes	Yes	Yes	Yes	Yes	Yes	Yes	Yes	100%	Low
Wu et al.	Yes	Yes	Yes	Yes	Yes	Yes	Yes	Yes	100%	Low
Zhou et al.	Yes	Yes	Yes	Yes	Yes	Yes	Yes	Yes	100%	Low
Frank et al.	Yes	Yes	Yes	Yes	Yes	Yes	Yes	Yes	100%	Low
Parvizi et al.	Yes	Yes	Yes	Yes	Yes	Yes	Yes	Yes	100%	Low
Asken et al.	Yes	Yes	Yes	Yes	Yes	Yes	Yes	Yes	100%	Low

Q1. Were the criteria for inclusion in the sample clearly defined?

Q2. Were the study subjects and the setting described in detail?

Q3. Was the exposure measured in a valid and reliable way?

Q4. Were objective, standard criteria used for the measurement of the condition?

Q5. Were confounding factors identified?

Q6. Were strategies to deal with confounding factors stated?

Q7. Were the outcomes measured in a valid and reliable way?

Q8. Was appropriate statistical analysis used?

**Table 3:** The JBI critical appraisal tool for cohort studies

Study	Q1	Q2	Q3	Q4	Q5	Q6	Q7	Q8	Q9	Yes %	Risk of bias
Mattsson et al.	Yes	Yes	Yes	Yes	Yes	Yes	Yes	Yes	Yes	100%	Low

Q1. Were the groups comparable other than the presence of disease in cases or the absence of disease in controls?    Q5. Was exposure measured in the same way for cases and controls?

Q2. Were cases and controls matched appropriately?

Q3. Were the same criteria used for the identification of cases and controls

Q4. Was exposure measured in a standard, valid and reliable way?

Q6. Were confounding factors identified?

Q7. Were strategies to deal with confounding factors stated?

Q8. Were outcomes assessed in a standard, valid and reliable way for cases and controls?

Q9. Was the exposure period of interest long enough to be meaningful?

Q10. Was appropriate statistical analysis used?

**Table 4:** The JBI critical appraisal tool for case–control studies

Study	Q1	Q2	Q3	Q4	Q5	Q6	Q7	Q8	Q9	Q10	Q11	Yes %	Risk of bias
Wang et al.	Yes	Yes	Yes	Yes	Yes	Yes	Yes	Yes	No	No	Yes	81%	Low

Q1. Were the two groups similar and recruited from the same population?

Q2. Were the exposures measured similarly to assign people to both exposed and unexposed groups?

Q3. Was the exposure measured in a valid and reliable way?

Q4. Were confounding factors identified?

Q5. Were strategies to deal with confounding factors stated?

Q6. Were the groups/participants free of the outcome at the start of the study (or at the moment of exposure)?

Q7. Were the outcomes measured in a valid and reliable way?

Q8. Was the follow-up time reported and sufficient to be long enough for outcomes to occur?

Q9. Was follow-up complete, and if not, were the reasons for loss to follow-up described and explored?

Q10. Were strategies to address incomplete follow-up utilized?

Q11. Was appropriate statistical analysis used?

### Heterogeneity of the data

**Fig. 5:**
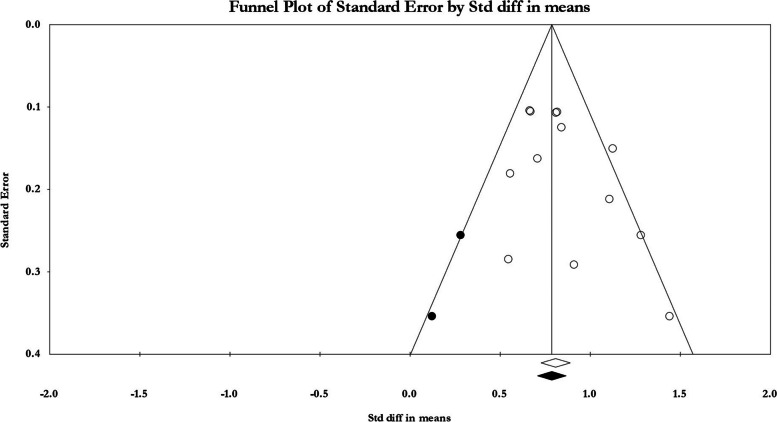
Publication bias. Funnel plot for NFL concentration in included studies

The prediction interval analysis revealed a mean effect size of 0.44, with a confidence interval of 0.36 to 0.51. Furthermore, it indicated that in 95% of comparable populations, the true effect size ranged from 0.17 to 0.70. The findings showed significant heterogeneity among the analyzed publications, as demonstrated by the prediction interval, which displayed a wider range of potential treatment outcomes compared to the confidence interval. The potential sources of heterogeneity may be associated with study design, patient characteristics, assay methods, or statistical approaches. The funnel plot analysis included 15 publications and showed a symmetric plot of standard error versus intervention effect, indicating no significant publication bias among the studies (see Fig. 5).

## Discussion

Our findings confirm that plasma NFL levels are significantly higher in AD patients than in MCI patients and healthy controls. The values are also higher in MCI patients than in healthy controls. The quality of the identified studies was found to be high based on JBI critical appraisal. However, the heterogeneity of the data was also shown to be high.

Two important issues were taken into account in this meta-analysis. First, our study evaluated the results emerging from publications that used plasma levels of NFL to differentiate between MCI, AD, and healthy controls. While the advantages of CSF biomarkers for AD diagnosis are well-known, there are also some disadvantages such as the invasive procedure required for obtaining CSF and inter-laboratory standardization and reproducibility of CSF biomarker measurements [33]. In contrast, blood biomarkers for AD are gaining attention because they are less invasive and easier to obtain than CSF biomarkers with the potential for earlier and more convenient detection of AD [34]. Additionally, blood samples can be stored and transported more easily than CSF samples, which must be analyzed within a few hours of collection. This allows for wider accessibility and feasibility of blood-based biomarker testing, especially in remote areas or regions with limited healthcare infrastructure. Furthermore, blood-based biomarker testing is generally more cost-effective compared to CSF-based testing, making it a more accessible option for patients [35].

In addition, due to the high cost and the unavailability of imaging methodes such as positron emission tomography (PET), and magnetic resonance imaging (MRI), detecting the blood biomarkers related to AD, offer greater convenience for simultaneously screening a large cohort of individuals [36].

Second, this meta-analysis only included studies that used SIMOA-based detection of NFL in plasma samples of MCI and AD patients and healthy controls. In the past, NFL was measured using ELISA and sensitive electro-chemiluminescence (ECL) methods. However, with the introduction of SIMOA, the measurement of NFL in blood samples became 125 and 26 times more sensitive than ELISA and ECL, respectively [9]. Besides, SIMOA is highly specific and can discriminate between closely related molecules with a high degree of accuracy. This method reduces the requirement for CSF collection and permits more frequent measurement due to the ease of obtaining blood samples [37]. This is important for the detection of complex biomarkers, such as those involved in neurodegenerative diseases, where subtle differences in biomarker isoforms can have diagnostic significance [38]. Also, SIMOA has a wide dynamic range and can measure biomarker levels over a large range of concentrations. This makes it possible to measure biomarker levels in both healthy and diseased populations, increasing the clinical utility of the technology [38].

Improving ultra-sensitive technologies has been crucial in propelling the field forward. SIMOA is a complex technology that requires specialized instrumentation and expertise for operation. From a research standpoint, SIMOA is the most well-established technology for detecting ultrasensitive blood-based biomarkers of AD pathology [39]. The process of translating blood-based biomarkers for AD into diagnostic biomarkers for regular use in patient care will require several stages. These stages include establishing a clear purpose, verifying the analytical and clinical performance within the intended use context, and obtaining regulatory authorization from entities [40].

The Simoa p-tau_181_ has been granted the Breakthrough Device designation by the FDA to assist in evaluating AD. Similarly, Simoa NfL has also received this designation for diagnosing multiple sclerosis. Moreover, certain companies provide NfL testing that is certified under the Laboratory Improvement Amendments (CLIA) [41]. Thus, there is an expectation that ultrasensitive methods like SIMOA will have great potential for clinical use in various clinical settings.

Currently, research studies have presented strong tests of blood markers to identify amyloid and tau pathologies that are unique to AD (Aβ peptides, p-tau). Additionally, there are also blood markers available for detecting non-specific neuronal (NFL, β-synuclein, ubiquitin-C-terminal-hydrolase-L1) and glial degeneration (glial fibrillary acidic protein) [10, 41, 42].

For instance, Shi et al. [43] utilized an ultrasensitive quantitative technique to illustrate the plasma concentrations of Aβ_40_, Aβ_42_, and NFL in individuals diagnosed with amnestic MCI (aMCI). In patients with aMCI, the levels of Aβ_40_ and Aβ_42_ were found to be decreased, whereas NFL levels were significantly elevated. Furthermore, increased plasma NFL concentrations were associated with reduced size of the hippocampus and total volume of gray matter in the left inferior and middle temporal gyrus. Similarly, Xiao et al. [44] assessed alterations in the levels of various plasma biomarkers, including Aβ_40_, Aβ_42_, t-Tau, NFL, and p-Tau_181_ in multiple stages of AD using SIMOA. As cognitive impairment progressed, levels of Aβ_40_, Aβ_42_ and Aβ_42_/Aβ_40_ decreased while levels of t-Tau, NFL and p-Tau_181_ increased. Among all plasma biomarkers, p-Tau181 was identified as a potential biomarker related to symptoms while NFL was considered a potential non-specific biomarker of neurodegeneration. In another study, Fortea et al. [45] assessed a population at high risk of AD and categorized the samples as asymptomatic, prodromal, or AD dementia. They measured Aβ_40_, Aβ_42_, t-Tau, p-Tau_181_, and NFL levels in both plasma and CSF using the SIMOA method. Plasma NFL levels were found to distinguish between the asymptomatic and prodromal groups as well as between the asymptomatic and dementia groups.

Plasma NFL levels are associated with cognitive deficits, progressive neural atrophy, and neurodegeneration in AD patients [46]. Elevated levels of plasma NFL were also appeared to be related with baseline CSF biomarkers including reduced Aβ_42_ and elevated total tau (t-Tau) and p-tau levels. Additionally, MRI measures, such as reduced hippocampal volumes, decreased regional cortical thickness, increased ventricular volumes, and reduced FDG-PET uptake were also associated with higher levels of plasma NFL in these patients [25, 46]. These findings were also generalizable to individuals with early-onset AD. In that light, Watson et al., found an increase in plasma NFL levels in AD patients with PSEN1 or APP mutations suggesting that NFL may assist as an initial diagnostic biomarker for early-onset AD, as plasma NFL concentrations were found to be elevated before the manifestation of symptoms (preclinical and prodromal AD) and corresponded with the severity stage of the disease [47]. Furthermore, in a prospective case–control study, Mattsson and colleagues found the precision of plasma NFL in distinguishing AD patients from healthy controls was equivalent to well-known CSF AD biomarkers and substantially higher than plasma tau [6]. These findings were duplicated in another study by Sugerman et al. where they showed that NFL is more accurate predictive factor in dementia than t-Tau, with the possibility to distinguish AD from MCI [21].

However, it’s important to note that a single plasma biomarker may not be sufficient to diagnose a disease; a combination of biomarkers could be more useful in distinguishing AD patients from healthy individuals.

Overall, SIMOA-based assay of plasma NFL levels is a promising approach in monitoring neurodegeneration in MCI and AD patients. It has the potential factor to provide less invasive and more economical alternative to current diagnostic methods. However, more research is needed to fully establish the diagnostic accuracy and clinical utility of this approach.

### Limitations

Our meta-analysis had several potential limitations. First, the studies we mainly included relied on clinical criteria for diagnosing AD patients, which may result in selection bias of participants, and more studies warranted to diagnose the patient via clinical assessment and brain imaging such as PET and MRI. Second, our access to data and papers was not universal, and we only searched PUBMED, SCOPUS, Web of Science, and google scholar, which could have influenced our results. Third, the studies we analyzed had limited sample sizes, potentially affecting accuracy; therefore, more multicenter studies with larger sample sizes are required. Fourth, our meta-analysis was restricted to studies published in English, so we may have missed relevant research in other languages, which may result in selection bias in our study.

## Conclusion

This meta-analysis demonstrated that by measuring NFL levels using the SIMOA method, we are able to differentiate AD and MCI patients from the healthy controls. In that light, NFL has the potential to be used as a diagnostic marker in AD patients. However, the included studies showed high heterogeneity, indicating that the outcome of this meta-analysis should be treated with caution.

## Data Availability

All data generated or analyzed in this work are included in the published version.
